# One-handed knot tying technique in single-incision laparoscopic surgery

**DOI:** 10.4103/0972-9941.72401

**Published:** 2011

**Authors:** John Thanakumar, Pravin Hector John

**Affiliations:** Department of Minimal Access and Bariatric Surgery, Global Hospital & Health City, 439, Cheran Nagar, Perumbakkam, Chennai, India

**Keywords:** Knot, single-incision laparoscopic surgery, surgery

## Abstract

In an open surgery, two-handed as well as one-handed knot tying is commonplace. Knot tying in laparoscopic surgery traditionally involves the use of two instruments (for fashioning an intracorporeal knot) or passing of a ligature around a tubular structure, exteriorising it, fashioning a knot, and sliding it down with a knot-pusher (external slip knot). With increasing interest in expanding applications of single-incision laparoscopic surgery (SILS), surgeons are faced with new challenges. In SILS it is not usually possible to utilise two instruments for knot tying as they lie almost parallel. We describe a novel one-handed knot tying technique devised specifically for use in SILS.

## INTRODUCTION

Ligation of a tubular structure is a fundamental step practised in surgery. In an open operation this is accomplished either with a length of suture tied using both hands or as a suture held in one hand, which is wrapped around a needle holder to fashion successive half knots. Laparoscopic knot tying is considered more complex as (a) it involves the use of two long instruments whose distant fulcrum poses certain ergonomic challenges, (b) the task needs to be performed by observing a two-dimensional image and (c) the cues obtained from the peripheral field of vision (as during open surgery) are absent when the endoscopic camera zooms in on the operative field.

Over the last few years, SILS is being utilised increasingly for a variety of procedures; single-incision laparoscopic cholecystectomy (SILC) is perhaps its most common application.[1,2] Almost all the reported series of SILC describe control of the cystic artery and cystic duct by using clips. With the increasing number of SILCs being undertaken, in a proportion of cases the surgeon is bound to encounter a wide cystic duct that will require ligation. We describe a novel one-handed knot tying technique devised specifically for use in SILS.

## TECHNIQUE

The following steps describe our technique in detail. For the purpose of clarity, the images have been obtained in an endotrainer using a length of stout tape used to fashion a one-handed knot around a length of tubing fixed horizontally. During execution of a one-handed knot during an SILC, the angled lie of the cystic duct, the limited space in the Calot’s triangle, the intermittent downward movement of the patient’s liver with breathing and the tendency of the end of the ligature to adhere to the tissue, pose additional challenges.

### Step 1: Encircling the duct

The prerequisite that facilitates one-handed ligation of a cystic duct is creation of a wide window above the duct by (a) freeing up the Hartmann’s pouch over a distance and (b) clipping and division of the cystic artery.

**Figure 1:**
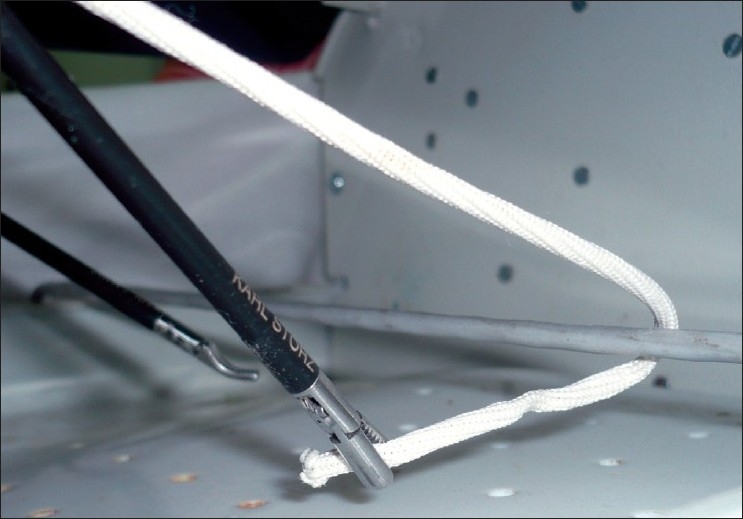
Suture material being passed around the tube

The length of the suture is shorter than that required for extracorporeal ligation and around 30 cm generally suffices. We prefer to use braided material such as 1-0 polyglactin (Vicryl, Johnson and Johnson, Mumbai, India) for its excellent knotting qualities. The tip of the suture is held with a needle holder and introduced through a port without a flap valve, to prevent it from getting caught. The suture is passed beyond the cystic duct, is re-grasped and brought around to form a loop [Figure 1]. The length of the suture passing beyond the duct should be around 8 – 10 cm, to allow formation of three throws of the knot.

### Step 2: Formation of an ‘O’ with over wrapping

**Figure 2:**
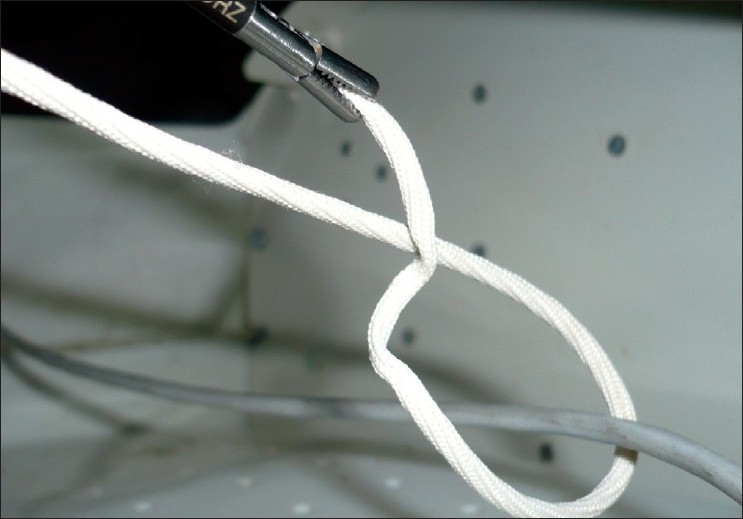
Formation of an ‘O’

Sufficient length of the suture is pulled around to form a loop in the shape of an ‘O’ with an over wrap [Figure 2]. At this stage the needle holder continues to hold the free end of the suture, while the surgeon holds the external end of the suture loosely in his non-dominant hand.

### Step 3: Formation of the first half knot

**Figure 3:**
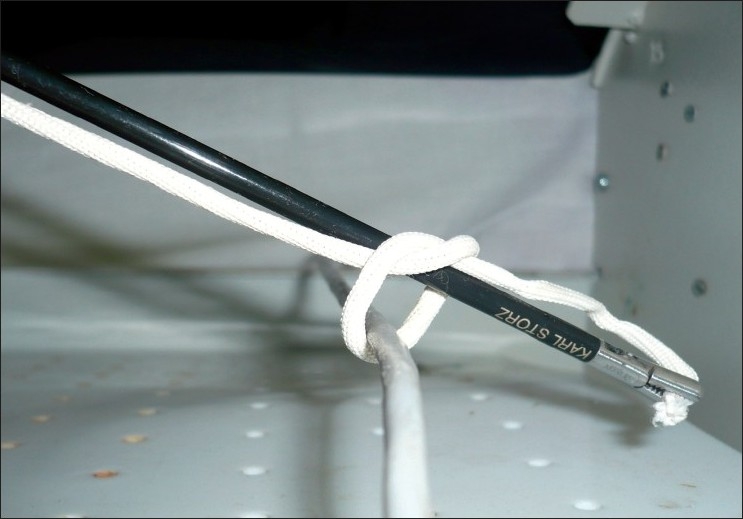
Grasper carrying the suture through the ‘O’ to initiate the first half knot

The grasper holding the end of the suture is passed through the ‘O’ thus inducing the formation of the first half knot [Figure 3]. At this stage, the surgeon starts to apply gentle traction on the external end of the suture.

### Step 4: Completion of the first half knot

**Figure 4:**
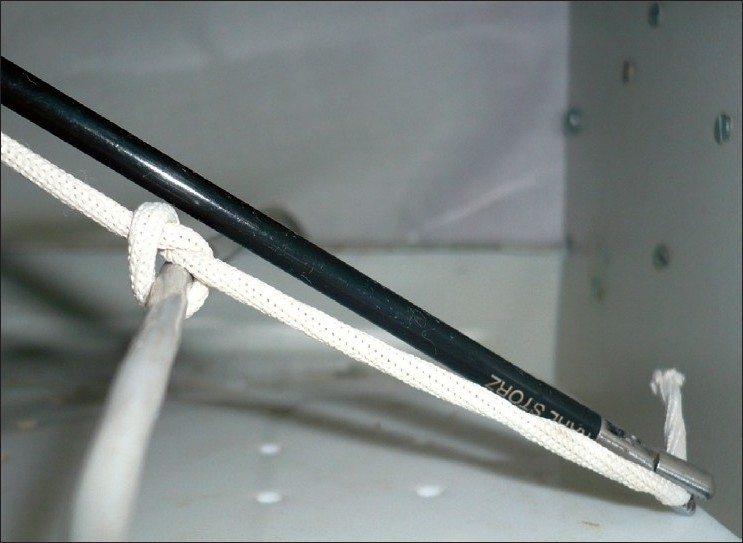
The first half knot is tightened into position

Once the ‘O’ is narrowed to a point where the first half knot will not unravel, the suture held in the grasper is released and the needle holder eased out of the ‘O’. The free end of the suture is re-grasped with the needle holder, traction is applied to the external end and with minor sideways movements of the tip of the needle holder the first half hitch is tightened precisely at the desired point [Figure 4]. This is easily remembered as conversion of the ‘O’ to a ‘Q’. The snugness of the knot is confirmed by observing the co-option of the walls of the structure being ligated.

### Step 5: Formation of the second ‘O’ by under wrapping

**Figure 5:**
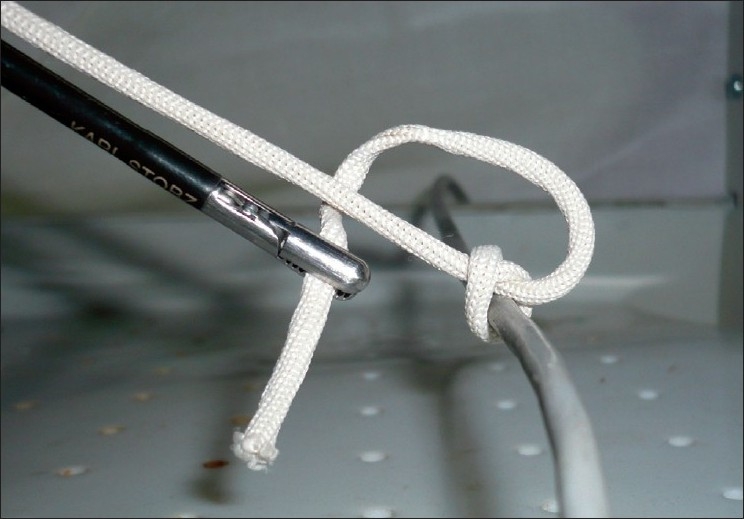
Formation of second ‘O’ by underwrapping.

At this stage the needle holder releases the tip and re-grasps the suture a few centimeters proximal to the tip. The needle holder is withdrawn and is rotated slightly to allow the short end of the suture to be under wrapped around the long end forming a second ‘O’ [Figure 5]. It is important that at this stage, the surgeon relaxes the traction on the long end so that the shape of ‘O’ is well formed.

### Step 6: Formation and completion of the second half knot

**Figure 6:**
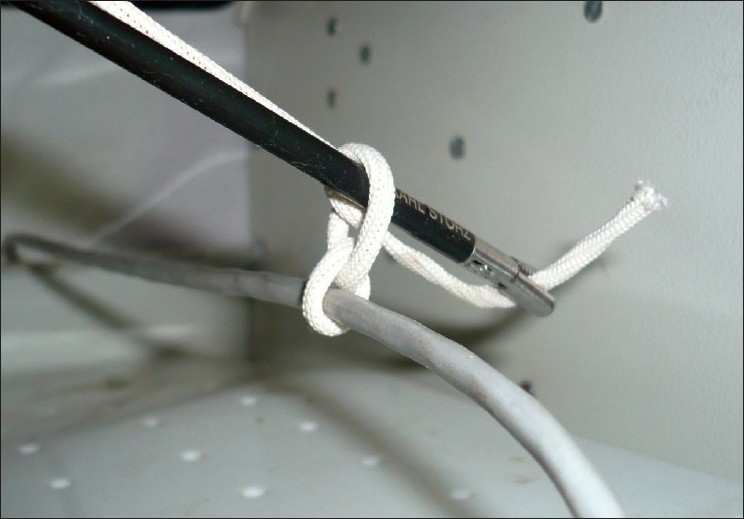
End of the suture carried through the ‘O’

**Figure 7:**
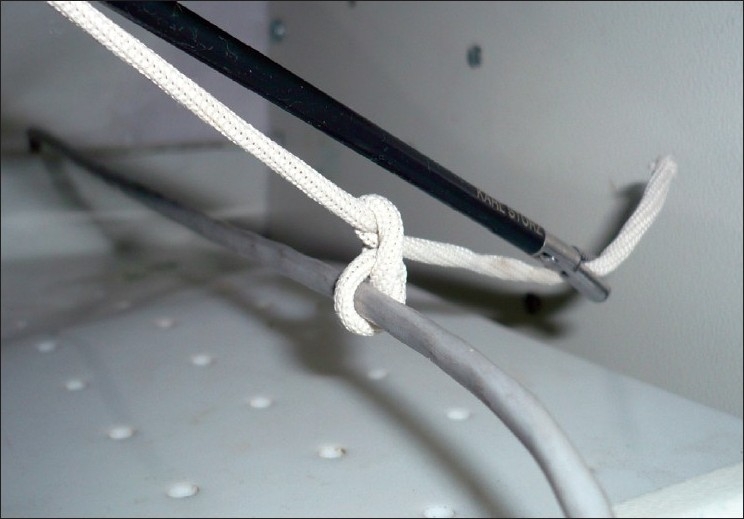
Completion of the knot by pulling the ends of the suture in opposite directions

The needle holder now pushes the tip of the suture through the ‘O’ as in step 3 [Figure 6]. Once again, the tip is released, the needle holder withdrawn from the loop and the tip re-grasped and pushed away, at the same time applying traction on the external end with the other hand. This manoeuvre tightens the second half knot into position, once again converting the second ‘O’ to a ‘Q’ [Figure 7].

An additional one or two half knots may be fashioned to complete the square knot.

## DISCUSSION

The knots used in laparoscopic surgery have been of two types – internal square knots[3] and external slip knots.[4] The former is tied using an intracorporeal technique and requires two instruments that fashion a knot using a length of ligature. In conventional laparoscopic surgery, the ports are so placed that the instruments introduced through them make an angle of between 45° and 70° with one another, thus facilitating intracorporeal knot tying. The external slip knots, on the other hand, are accomplished ‘extracorporeally’ by introducing a long length of suture via a port, passing it around the structure to be ligated, exteriorising the suture and fashioning a knot, which is then slid down using a knot-pusher. The safety of both these types of knots has been well-documented.[5,6]

Performance of a SILC poses peculiar challenges in terms of control of the pedicle. In the ‘puppeteer’ technique, the gallbladder is suspended / manipulated with the use of two transabdominal sutures and only one instrument is available for dissection and ligation.[1] Even when two instruments are used for performing SILC, two-handed knot-tying becomes almost impossible as the instruments lie almost parallel to one another. The problem may be overcome by using one or even two roticulating instruments so that the tips of the instruments meet at an angle and facilitate knot tying. No reports of such techniques are available as yet.

A one-handed knot refers to a knot tied with one end of the suture kept fixed in one hand as the second hand carries out the manoeuvers necessary to form the knot. In open surgery, a square knot can be tied easily using this technique. In laparoscopic surgery, the use of a single-handed knot has been described in only two reports previously. Ieong *et al*. described a one-handed knot for closure of the internal ring during laparoscopic repair of inguinal hernia in children.[7] They passed the needle through the defect and secured it within the anterior abdominal wall. The tail of the suture was passed backwards to the bight, ensuring that the bight was under tension. The laparoscopic instrument was then passed through the loop and the tail was pulled through, thus forming a square knot. The step was repeated to form multiple throws, which were snugged down. Also the technique of single-handed knot tying has been described by Ou *et al*. for ligation of the renal pedicle during hand-assisted retroperitoneoscopic nephrectomy.[8] In their technique the index finger of the non-dominant intra-abdominal hand (IAH) encircled the renal vessel and a suture introduced through a laparoscopic port was passed around it. Fingers of the IAH and the outside hand holding the end of the suture were used in tandem to form knots, which were snugged down with the index finger of the IAH.

The one-handed knot tying technique described by us is particularly helpful in the ligation of a wide cystic duct, during SILC. We have used it on over 25 cases and found it to be secure. The advantage of this technique is that it does not entail the use of any instruments other than those available in a standard laparoscopic set. Ligation of a wide cystic duct using the one-handed knot tying technique is not only cost-effective, but it also allows the surgeon to carry out the entire surgery without the use of any ancilliary ports placed away from the umbilicus. Nevertheless, as with traditional multi-port LC the surgeon should not hesitate to place an additional port for completing the knot safely if the one-handed knot tying is not possible. One-handed knot tying can be learnt by understanding the principles of fashioning the knot and practising it in an endotrainer. Attention to slow and purposeful choreography forms the key to mastering it. It is hoped that this simple and convenient knot tying technique will not only find a place in the armamentarium of the surgeon performing SILS, but also become applicable in conventional laparoscopic as well as in hybrid techniques combining laparoscopic and natural orifice translumenal endoscopic surgery (NOTES).
